# Association of Exclusive Breastfeeding with Asthma Risk among Preschool Children: An Analysis of National Health and Nutrition Examination Survey Data, 1999 to 2014

**DOI:** 10.3390/nu14204250

**Published:** 2022-10-12

**Authors:** Chi-Nien Chen, Yu-Chen Lin, Shau-Ru Ho, Chun-Min Fu, An-Kuo Chou, Yao-Hsu Yang

**Affiliations:** 1 Department of Pediatrics, National Taiwan University Hospital Hsin-Chu Branch, Hsinchu 30059, Taiwan; 2 Department of Pediatrics, National Taiwan University College of Medicine, Taipei 10051, Taiwan; 3 Department of Pediatrics, National Taiwan University Children’s Hospital, Taipei 10041, Taiwan

**Keywords:** breastfeeding, breastmilk, pediatric asthma, allergy, cohort study

## Abstract

Breastmilk contains many important nutrients, anti-inflammatory agents, and immunomodulators. It is the preferred nutrition source for infants. However, the association of the duration of exclusive breastmilk feeding (BMF) with asthma development is unclear. Data on children from the United States who participated in the National Health and Nutrition Examination Survey (NHANES) from 1999 to 2014 were obtained. We examined the association between the duration of exclusive BMF and asthma in 6000 children (3 to 6 years old). After calculating the duration of exclusive breastfeeding according to answers to NHANES questionnaires, the estimated duration of exclusive BMF was divided into five categories: never breastfed or BMF for 0 to 2 months after birth; BMF for 2 to 4 months after birth; BMF for 4 to 6 months after birth; and BMF for ≥6 months after birth. The overall prevalence of asthma in children aged 3 to 6 years was approximately 13.9%**.** The risk of asthma was lower in children with an exclusive BMF duration of 4 to 6 months (aOR, 0.69; 95% CI, 0.48–0.98), after adjustment for potentially confounding factors. Subgroup analysis revealed that children of younger ages (3 to 4 years old) benefited most from the protective effects of exclusive BMF for 4 to 6 months (aOR, 0.47; 95% CI, 0.27, 0.8). We found that exclusive BMF, especially BMF for 4 to 6 months, is associated with a decreased risk of asthma in preschool-age children. The protective effect appeared to be diminished in older children. The potential mechanism needs further investigation.

## 1. Introduction

Asthma is the most common chronic disease in childhood, affecting 8.4% of children in the United States [1]. Most children who suffer from asthma experience their first attack when they are of preschool age, and asthma is a major cause of hospitalization and emergency room visits by preschool children. Asthma prevalence has increased in recent decades [2,3]. It is a chronic airway inflammatory disease, and lung development is a complex process that begins during the intrauterine period and extends through early infancy after birth [4]. Therefore, a beneficial strategy to prevent the development of pediatric asthma is needed, and such a strategy should focus on children’s early postnatal period.

Breastfeeding is recognized as having many benefits for short-term and long-term health, including decreasing the risks of upper airway infection, gastrointestinal infection, obesity, and diabetes [5]. From a cost perspective, an increase in the rate of breastfeeding could save billions of dollars and improve maternal and child health [6,7]. The World Health Organization (WHO) and the American Academy of Pediatrics (AAP) recommended that infants should be exclusively breastfed for 6 months, due to the advantages of breastfeeding [8,9]. Breastmilk contains some unique substances that have positive biological effects on the regulation of immune function and the promotion of lung growth. Exclusive breastfeeding for more than 4 months could improve lung growth and lung function in children [10,11]. Accordingly, breastfeeding is considered to have protective effects against the development of asthma.

Some potential mechanisms may explain this association, including microbiome composition, bioactive factors in breastmilk, and the epigenetic effects of breastmilk. Several epidemiologic studies have shown the protective effect of breastfeeding against pediatric asthma [12,13]. Recently, a meta-analysis by Harvey et al. [14] reported a 55% decrease in the risk of wheeze-related outcomes among breastfed children, compared with children who were never breastfed during early childhood. However, some other studies have reported inconsistent results regarding the protective effect of breastfeeding [15,16]. Another meta-analysis study on childhood asthma found that longer durations of breastfeeding and exclusive breastfeeding may only provide a protective effect for pre-school age children [17]. Whether breastfeeding protects against asthma in early childhood and/or for more extended periods is controversial. However, performing a randomized controlled trial of infant feeding practices to clarify this relationship was not practicable.

Therefore, we hypothesized that a longer duration of exclusive breastmilk feeding (BMF) may be associated with a decreased risk of asthma in early childhood. To test this hypothesis, we examined the relationship between BMF and asthma in a nationally representative sample of children in the United States, using data from the National Health and Nutrition Examination Survey (NHANES) from 1999 to 2014.

## 2. Materials and Methods

The NHANES comprises a series of stratified and multistage surveys designed to investigate the health and nutritional status of adults and children in the United States The survey enrolls a nationally representative sample of approximately 5000 participants each year. A standardized questionnaire is used to collect data on demographic variables, dietary behavior, medical conditions, health insurance, and early childhood conditions for further investigation. The data collection and survey protocols are described elsewhere [18,19,20]. For the 1999–2000 to 2013–2014 surveys, the overall response rates ranged from 71% to 84%. 

Demographic variables included age, sex, and race/ethnicity. Poverty-to-income ratio (PIR) and insurance coverage data were collected from standardized demographic questionnaires. Race/ethnicity was categorized as Hispanic, non-Hispanic White, non-Hispanic Black, and other (including multiracial) (see Table 1). The PIR was categorized as <1.85 or ≥1.85, as described in previous research [21]. 

Our study was approved by the Institute Review Board (IRB) of National Taiwan University Hospital’s Hsin-Chu Branch (IRB No. 107-089-E).

### 2.1. Breastmilk Exposure Assessment

Exclusive BMF duration was estimated on the basis of dietary and behavioral questionnaires targeting children aged 3 to 6 years. In the NHANES surveys from 1999 to 2000 to 2007 to 2008, the duration of exclusive BMF was assessed by the questions set out below and categorized as follows: “Never”; “<2 months”; “>=2 to <4 months”; “>=4 to <6 months”; and “>=6 months”. The categories were determined by calculating the duration of exclusive breastfeeding according to answers provided to the following questions in the questionnaires.

*“DBQ010- Was ever breastfed or fed breast milk?”*

*“DBD030- How old was SP when he/she completely stopped breastfeeding or being fed breast milk?”*

*“DBD040- How old was (SP) when he/she was first fed formula on a daily basis?”*

In the NHANES surveys from 2009–2010 to 2013–2014, the duration of exclusive BMF was assessed by the questions below:

*“DBQ010- Was ever breastfed or fed breast milk?”*

*“DBD030- How old was SP when he/she completely stopped breastfeeding or being fed breast milk?”*

*“DBD041- How old was (SP) when he/she was first fed formula?”*

### 2.2. Asthma Outcome Assessment

Among preschool-age children, a diagnosis of asthma may be uncertain because conventional pulmonary function tests are not able to confirm the diagnosis. Therefore, the diagnosis of asthma in early childhood is mainly based on clinical judgment. In our study, the diagnosis of asthma was defined by a response of “*Yes*” to the question: *“Has a doctor or other health professional ever told you/SP that you have asthma?”* We restricted our study to subjects over three years of age, primarily to reduce the overdiagnosis of early childhood asthma. Because a viral infection mainly causes a wheezing illness in children of younger ages, such an infection is often mistaken for asthma due to such early wheezing symptoms.

### 2.3. Statistical Analysis

Descriptive analyses were performed using the PROC SURVEYMEANS procedure to account for the survey’s complex weighting factors. To explore the association of exclusive breastmilk feeding duration with the risk of asthma, we performed logistic regression analyses using the PROC SURVEYLOGISTIC procedure. Multivariable models, including variables such as age, sex, race/ethnicity, family PIR, maternal age at delivery, family smoking exposure, low birth weight, survey cycle, and insurance coverage, were assessed. Participants with missing data for these variables were excluded from complete data analysis. Crude odds ratios (ORs), adjusted ORs, and 95% confidence intervals (CIs) were estimated. All reported *p* values < 0.05 were considered significant. Statistical analyses were performed using SAS statistical software (version 9.4, SAS Institute Inc., Cary, NC, USA).

## 3. Results

**Figure 1:**
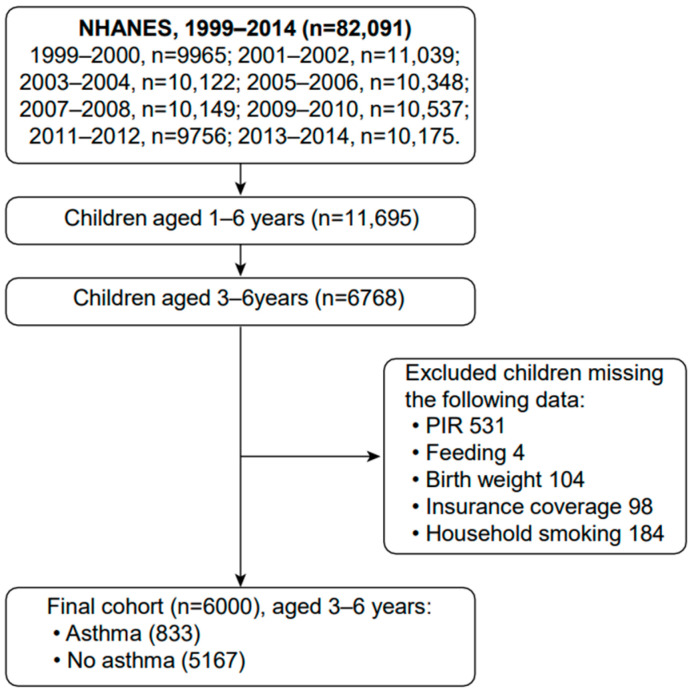
Flow chart of study population.

A total of 82,091 participants were enrolled in the NHANES between 1999 and 2014. Among them, 6768 were children aged 3 to 6 years. After excluding children with missing data, a total of 6000 children aged 3 to 6 years were analyzed in our cohort study (Figure 1). The baseline characteristics of the study participants are listed in Table 1. Trends in exclusive breastfeeding duration and asthma are presented in Table 2.

Table 2 summarizes the associations between different exclusive breastfeeding durations and asthma, with crude and adjusted ORs and 95% CIs after adjustment for potential confounding factors. The aORs associated with exclusive breastfeeding durations of 4 to 6 months and more than 6 months compared with “Never” were 0.69 (95% CI, 0.48 to 0.98) and 0.9 (95% CI, 0.62 to 1.3), respectively.

**Figure 2:**
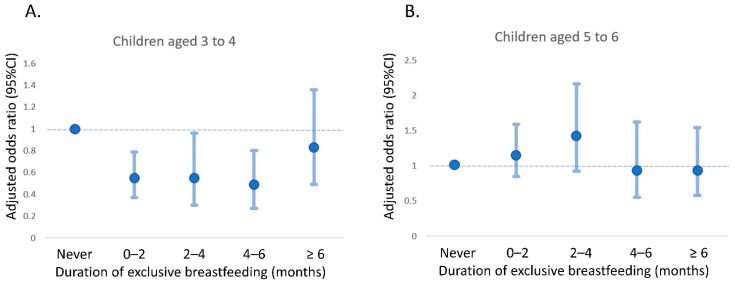
Association of exclusive breastmilk feeding duration with risk of asthma by age. (**A**) children aged 3 to 4 months; (**B**) children aged 5 to 6 months.

Subgroup analysis was performed to determine the effects on different age groups. No statistically significant association was found between BMF duration and asthma development in children aged 5 to 6 years (Table 3). Among children aged 3 to 4 years, the aORs associated with exclusive breastfeeding for 4 to 6 months and more than 6 months compared with “Never” were 0.47 (95% CI, 0.27 to 0.8) and 0.82 (95% CI, 0.49 to 1.36), respectively. However, for children aged 5 to 6 years, the aORs associated with exclusive breastfeeding for 4 to 6 months and more than 6 months, compared with “Never,” were 0.94 (95% CI, 0.55 to 1.62) and 0.94 (95% CI, 0.58 to 1.54), respectively. Figure 2 illustrates the relationship between exclusive breastfeeding duration and asthma risk by age. A U-shaped relationship between exclusive breastfeeding duration and the risk of asthma in children aged 3 to 4 (Figure 2A) and a duration of 4 to 6 months was associated with the lowest risk of asthma among them.

## 4. Discussion

We analyzed the data of a nationally representative sample of American children from 1999 to 2014 and found that, for preschool children, exclusive breastfeeding for 4 to 6 months was associated with a lower risk of developing asthma than never breastfeeding. The main findings suggest that the protective effect of breastfeeding is strongest at a young age (3 to 4 years old).

Our study’s finding was in line with that of a previous Korean cohort study by Kim et al. [22], who found that exclusive breastfeeding for more than 6 months did not have a statistically significant effect on the development of asthma. This finding contrasts with that of a study from Japan that concluded that exclusive breastfeeding for 6 to 7 months was associated with a lower risk of hospitalization for asthma [23]. In addition, three different systematic reviews and meta-analyses that focused on breastmilk and asthma or childhood wheezing reported the existence of heterogeneity among the studies [24,25,26]. Possible explanations for the conflicting evidence may be different study designs or inconsistent definitions of breastfeeding and asthma outcomes. Whether breastfeeding can decrease the risk of asthma is controversial. Some studies found that breastfeeding did not have a protective effect against the development of asthma [15,16]. The main reason for the inconsistent findings among previous studies may be variations in study methods and definitions of breastfeeding. Some researchers analyzed exposure to breastfeeding as “ever” or “never”, and some analyzed the duration of breastmilk feeding. Different definitions and exposure measurements likely require different outcome assessments to clarify the true effect of BMF on the development of asthma. Therefore, we precisely defined breastmilk exposure as the exclusive duration of breastmilk feeding, to evaluate the relationship between infant feeding practices and subsequent asthma risk. In addition, as it is quite difficult to diagnose asthma in younger children, a clear definition and diagnosis of asthma was needed to allow comparisons between different studies and to reach a consensus standard [27].

An interesting finding of our study was that the protective effect against asthma disappeared with exclusive breastfeeding for more than six months. An explanation may be that maternal or family history of asthma may determine the initiation and duration of BMF, indicating that babies with an increased risk of asthma based on family history may have been more likely to be breastfed longer than those without such a family history. These decisions may neutralize the protective effects of breastfeeding on asthma among children with an increased risk of asthma due to family history [28,29].

### 4.1. Interpretation of the Subgroup Analysis by Age

Breastfeeding does not seem to be very effective in protecting older children against asthma. Our results demonstrated that exclusive breastfeeding for 4 to 6 months had the most protective effect against asthma development in preschool children aged 3 to 4 years; this effect was not observed in children aged 5 to 6 years. This may be related to the development of asthma caused by environmental factors. Indoor and outdoor air pollutants, biological allergens, and environmental chemicals can cause pre-existing airway inflammation exacerbation, leading to airway hyperresponsiveness and asthma after cumulative exposures during aging [30,31]. Other explanations may be related to the allergic march and the diagnosis of asthma. Early asthmatic wheezing and other minor symptoms may start in early childhood. These symptoms may persist or deteriorate throughout school age and adolescence [32]. The diagnosis of asthma using lung function tests cannot be performed in young children, due to the limited cooperation by young children and suboptimal forced exhalation maneuvers [33]. The diagnosis of asthma is more accurate in older children. Therefore, we restricted the primary analysis group to children over three years of age to reduce the impact of the difficulty in diagnosing asthma in children at younger ages.

### 4.2. Mechanisms Driving the Protective Effect of BMF against Asthma

Breastfeeding may influence epigenetic processes through global DNA methylation patterns [34,35]. Although many epigenetic mechanisms for breastfeeding and epigenetic processes have been postulated, further well-designed studies should be performed to eliminate confounding effects. Possible confounding factors may include maternal prepregnancy factors (such as maternal age, parity, body mass index, and pre-existing conditions), gestational factors (drug exposure, tobacco use, and delivery mode), and socioeconomic factors (educational attainment, occupation, and family income) [36].

Immunomodulatory constituents and anti-inflammatory agents in breastmilk are important for the prevention of childhood asthma. Breastfeeding plays a key role in the development of oral and intestinal microbiota in infants. Breastmilk provides pioneering species and aids in the establishment of the final intestinal microbiota composition. Human milk oligosaccharides (HMOs) also have the ability to influence the development of the immune system [37]. Most HMOs are indigestible in newborn children and have a prebiotic effect. In the lower intestinal tract, HMOs provide substrates for gut bacteria and are especially utilized by Bacteroides and Bifidobacteria [38]. A predominance of these intestinal bacteria might prevent the proliferation of pathogenic bacteria, improve gut barrier function, and mitigate the risks of severe intestinal infections. It has been proposed that gut microbiota alterations in early childhood can change immunological tolerance and predispose individuals to allergic disorders, including asthma [39].

Some studies have proven that specific HMOs can regulate the immune response directly via viral pathogen mitigation and regulation of host immune cells. In addition, there is another possible mechanism by which HMOs protect against asthma. Approximately 1% of HMOs are absorbed into the blood circulation and finally reach end organs, such as the lungs [40]. HMOs can affect the turnover of airway epithelial cells and the formation of glycocalyx mucus, and interact with immune cells and pathogens to protect against asthma [41].

### 4.3. Strengths and Limitations

This study had several strengths. First, we used data from a large nationally representative cohort for analysis, and the results summarize the trends of BMF practices and their effects on asthma outcomes over the course of 16 years. We assessed the protective effect of BMF while controlling for multiple confounding factors. Performing a randomized controlled trial of infant feeding practices would not have been practicable; therefore, we provided beneficial evidence of the association by assessing the dose-response effects of BMF stratified by exclusive BMF duration.

This study had some limitations. First, this was a cross-sectional study, and the study design could not fully eliminate the possibility of reverse causation. Participants with a longer duration of exclusive BMF may have had early allergic or wheezing symptoms and signs, and mothers may have intentionally prolonged the duration of breastfeeding. Some of the mothers may have practiced exclusive BMF to promote better offspring outcomes. There were no specific questionnaire questions regarding an association between the timing of wheezing or allergic symptoms and the age of asthma onset. Without a time-sequence evaluation, it was difficult to identify this association clearly. Second, because of the study design, this study could not differentiate between the effects of direct breastfeeding and feeding with expressed breastmilk. Direct breastfeeding may have better protective effects than other modes of BMF [12]. There have been various definitions of breastmilk exposure among different studies, which may have influenced the outcomes, resulting in the uncertain benefit of BMF. Third, recall bias regarding BMF duration should be considered [42]. Inaccuracies in the recall of BMF may have led to the reporting of shorter or longer BMF durations, especially for those children of older ages. We used the NHANES database questions to estimate the duration of exclusive breastfeeding. There may be other diets that could not be fully captured by the database questions, potentially leading to biased estimates of the duration of exclusive breastfeeding. Accordingly, the results should be interpreted carefully. Fortunately, the impact of recall bias may be less than expected, because overall BMF durations were similar in other surveys [43]. Fourth, family medical history and environmental factors may increase children’s asthma risk. For example, a family history of allergic diseases, pets in the family home, and/or living in an urban or rural area are all potential influencing factors [44,45,46]. Researchers should consider such factors in future analysis. Unfortunately, in the NHANES database, such variables were not completely accounted for or were not available for a public study. Future research needs to investigate whether such factors, other than breastmilk itself, have substantial effects on children’s asthma.

In the future, a prospective cohort study is needed to clarify the temporal issues associated with potential reverse causation and more comprehensive data, such as data on the duration and types of breastfeeding, are needed to develop strategies to protect against asthma development.

## 5. Conclusions

In conclusion, we demonstrated a U-shaped relationship between exclusive breastfeeding duration and the risk of asthma in children aged 3 to 4 years, and a duration of 4 to 6 months was associated with a lower risk of asthma in preschool children, especially younger children, based on data from the National Health and Nutrition Examination Survey (NHANES) from 1999 to 2014. Public health policies to promote exclusive breastmilk feeding (BMF) may help to prevent the development of asthma in early childhood. Future studies are needed to investigate reverse causation and to elucidate the mechanism and interactions with potential confounding factors.

## Figures and Tables

**Table 1:** Clinical characteristics of the study participants and univariate analysis of the risk of asthma.

Characteristics		Total		Ever Asthma			
		N = 6000		Yes (n = 833)		No (n = 5167)	
		n	%	n	%	n	%
Age:	3–4 y	3110	51.8	391	46.9	2719	52.6
	5–6 y	2890	48.2	442	53.1	2448	47.4
Sex: male		3060	51	502	60.3	2558	49.5
female		2940	49	331	39.7	2609	50.5
Race: Mexican-American		1581	26.3	135	16.2	1446	28
	Other-Hispanic	497	8.3	85	10.2	412	8
	Non-Hispanic White	1772	29.5	204	24.5	1568	30.3
	Non-Hispanic Black	1600	26.7	322	38.7	1278	24.7
	Other/Multiracial	550	9.2	87	10.4	463	9
Poverty-to-income ratio:	<1.85	3710	61.8	537	64.5	3173	61.4
	≥1.85	2290	38.2	296	35.5	1994	38.6
Maternal smoking: yes		768	12.8	134	16.1	634	12.3
no		5232	87.2	699	83.9	4533	87.7
Insurance coverage: yes		5389	89.8	778	93.4	4611	89.2
no		611	10.2	55	6.6	556	10.8
Low birth rate: yes		590	9.8	124	14.9	466	9
no		5410	90.2	709	85.1	4701	91
Maternal age: ≥35 y		1291	21.5	180	21.6	1111	21.5
<35 y		4709	78.5	653	78.4	4056	78.5
Household smoking:		949	15.8	152	18.2	797	15.4
Survey Cycle	1999–2000	593	9.9	96	11.5	497	9.6
	2001–2002	745	12.4	96	11.5	649	12.6
	2003–2004	716	11.9	100	12	616	11.9
	2005–2006	831	13.9	114	13.7	717	13.9
	2007–2008	720	12	103	12.4	617	11.9
	2009–2010	751	12.5	101	12.1	650	12.6
	2011–2012	818	13.6	117	14.1	701	13.6
	2013–2014	826	13.8	106	12.7	720	13.9

**Table 2:** Association of breastmilk feeding duration with risk of asthma.

Duration	Total	Asthma	Prevalence	Crude OR	95% CI	Adjusted OR *	95% CI
Never	2140	358	16.73	1	Ref.	1	Ref.
0–2 m	1692	215	12.71	0.69	0.55–0.86	0.82	0.65–1.04
2–4 m	715	97	13.57	0.78	0.56–1.09	0.93	0.64–1.34
4–6 m	649	66	10.17	0.52	0.37–0.73	0.69	0.48–0.98
≥6 m	804	97	12.06	0.74	0.51–1.07	0.9	0.62–1.3

* Adjusted for age, sex, race, poverty-to-income ratio, maternal age at delivery, maternal smoking during pregnancy, low birth weight, household smoking exposure, insurance coverage, and survey cycle.

**Table 3:** Association of exclusive breastmilk feeding duration with risk of asthma by age.

	3–4 y					5–6 y				
Duration	N	Asthma	Prevalence	aOR *	95% CI	N	Asthma	Prevalence	aOR *	95% CI
Never	1075	188	17.49	1	Ref.	1065	170	15.96	1	Ref.
0–2 m	934	100	10.71	0.54	0.37–0.79	758	115	15.17	1.16	0.85–1.59
2–4 m	356	33	9.27	0.54	0.3–0.96	359	64	17.83	1.41	0.92–2.16
4–6 m	347	24	6.92	0.47	0.27–0.8	302	42	13.91	0.94	0.55–1.62
≥6 m	398	46	11.56	0.82	0.49–1.36	406	51	12.56	0.94	0.58–1.54

* Adjusted for sex, race, poverty-to-income ratio, maternal age at delivery, maternal smoking during pregnancy, low birth weight, household smoking exposure, insurance coverage and survey cycle.

## Data Availability

All information is available on the NHANES website.
